# Psoriatic skin inflammation induces a pre-diabetic phenotype via the endocrine actions of skin secretome

**DOI:** 10.1016/j.molmet.2020.101047

**Published:** 2020-06-26

**Authors:** Elizabeth A. Evans, Sophie R. Sayers, Xenia Kodji, Yue Xia, Mahum Shaikh, Alizah Rizvi, James Frame, Susan D. Brain, Michael P. Philpott, Rosalind F. Hannen, Paul W. Caton

**Affiliations:** 1 Department of Diabetes, School of Life Course Sciences, King's College London, UK; 2 Section of Vascular Biology & Inflammation, School of Cardiovascular Medicine & Sciences, BHF Centre for Cardiovascular Sciences, King's College London, London, UK; 3 A∗STAR - Agency for Science, Technology and Research - SRIS, Singapore; 4 Anglia-Ruskin University, Chelmsford, Essex, UK; 5 Springfield Hospital, Chelmsford, UK; 6 Centre for Cell Biology and Cutaneous Research, Blizard Institute, Queen Mary University of London, London, UK

**Keywords:** Psoriasis, Diabetes, Inflammation, Skin, Pancreatic islets, Adipose tissue

## Abstract

**Objective:**

Psoriasis is a chronic inflammatory skin disease that is thought to affect ∼2% of the global population. Psoriasis has been associated with ∼30% increased risk of developing type 2 diabetes (T2D), with numerous studies reporting that psoriasis is an independent risk-factor for T2D, separate from underlying obesity. Separately, studies of skin-specific transgenic mice have reported altered whole-body glucose homeostasis in these models. These studies imply a direct role for skin inflammation and dysfunction in mediating the onset of T2D in psoriasis patients, potentially via the endocrine effects of the skin secretome on key metabolic tissues. We used a combination of *in vivo* and *ex vivo* mouse models and *ex vivo* human imiquimod (IMQ) models to investigate the effects of psoriasis-mediated changes in the skin secretome on whole-body metabolic function.

**Methods:**

To induce psoriatic skin inflammation, mice were topically administered 75 mg of 5% IMQ cream (or Vaseline control) to a shaved dorsal region for 4 consecutive days. On day 5, mice were fasted for glucose and insulin tolerance testing, or sacrificed in the fed state with blood and tissues collected for analysis. To determine effects of the skin secretome, mouse skin was collected at day 5 from IMQ mice and cultured for 24 h. Conditioned media (CM) was collected and used 1:1 with fresh media to treat mouse explant subcutaneous adipose tissue (sAT) and isolated pancreatic islets. For human CM experiments, human skin was exposed to 5% IMQ cream for 20 min, *ex vivo*, to induce a psoriatic phenotype, then cultured for 24 h. CM was collected, combined 1:1 with fresh media and used to treat human sAT *ex vivo*. Markers of tissue inflammation and metabolic function were determined by qPCR. Beta cell function in isolated islets was measured by dynamic insulin secretion. Beta-cell proliferation was determined by measurement of Ki67 immunofluorescence histochemistry and BrDU uptake, whilst islet apoptosis was assessed by caspase 3/7 activity. All data is expressed as mean ± SEM.

**Results:**

Topical treatment with IMQ induced a psoriatic-like phenotype in mouse skin, evidenced by thickening, erythema and inflammation of the skin. Topical IMQ treatment induced inflammation and signs of metabolic dysfunction in sub-cutaneous and epidydimal adipose tissue, liver, skeletal muscle and gut tissue. However, consistent with islet compensation and a pre-diabetic phenotype, IMQ mice displayed improved glucose tolerance, increased insulin and c-peptide response to glucose, and increased beta cell proliferation. Treatment of sAT with psoriatic mouse or human skin-CM replicated the *in vivo* phenotype, leading to increased inflammation and metabolic dysfunction in mouse and human sAT. Treatment of pancreatic islets with psoriatic mouse skin-CM induced increases in beta-proliferation and apoptosis, thus partially replicating the *in vivo* phenotype.

**Conclusions:**

Psoriasis-like skin inflammation induces a pre-diabetic phenotype, characterised by tissue inflammation and markers of metabolic dysfunction, together with islet compensation in mice. The *in vivo* phenotype is partially replicated by exposure of sAT and pancreatic islets to psoriatic-skin conditioned media. These results support the hypothesis that psoriatic skin inflammation, potentially via the endocrine actions of the skin secretome, may constitute a novel pathophysiological pathway mediating the development of T2D.

## Highlights

• Topical IMQ treatment induced a psoriatic-like phenotype in mouse skin.
• Skin IMQ treatment induced inflammation and signs of metabolic dysfunction in sub-cutaneous and epidydimal adipose tissue, liver, skeletal muscle and gut tissue.
• Skin IMQ treatment induced islet compensation characterised by increased insulin and c-peptide response to glucose, and increased beta cell proliferation.
• The *in vivo* IMQ mouse phenotype is partially replicated by exposure of sAT and pancreatic islets to psoriatic-skin conditioned media.
• Psoriatic skin inflammation, potentially through the endocrine actions of the skin secretome, may constitute a novel pathophysiological pathway mediating the development of T2D.

## 1 Introduction

Psoriasis is a chronic inflammatory skin disease that is thought to affect ∼2% of the global population, with prevalence ranging from 8.5% in Norway to 0.91% in the USA [1,2]. The pathogenesis of psoriasis predominantly involves dysregulated Th17 pathway-mediated inflammatory response, leading to immune cell infiltration, epidermal hyperplasia through keratinocyte proliferation, and inflammation at lesional sites [3,4]. Clinical and epidemiological studies have demonstrated an association between psoriasis and a range of co-morbidities including type 2 diabetes (T2D) [[5], [6], [7]]. For example, psoriasis has been associated with ∼30% increased risk of developing T2D [7]. Patients with comorbid psoriasis diagnosed with T2D were younger at the age of diagnosis than non-psoriasis patients [8]. Intriguingly, studies have reported that psoriasis is an independent risk-factor for T2D and insulin resistance, separate from underlying obesity, with the severity of psoriasis correlating with increased likelihood of developing T2D and the requirement of insulin therapy [5,8]. These studies imply a direct role for skin inflammation in mediating the onset of psoriatic comorbidities. In support of this, keratinocyte-specific *SCD1* KO mice display altered glucose and lipid homeostasis [9], potentially via the endocrine actions of skin-derived IL6 [10], supporting a direct role for the skin in regulating whole-body metabolic homeostasis.

The mechanisms underlying the effects of skin inflammation on whole-body metabolism remain undetermined; however, the skin secretome may play a role. The skin secretes a wide range of proteins, lipids and small molecules [3,[11], [12], [13], [14], [15], [16]] with potential paracrine and endocrine effects. Many skin-derived factors, such as cytokines and glucocorticoids, have established metabolic functions, and altered expression and secretion of such factors would be predicted to exert metabolic effects [14,[18], [19], [20], [21], [22]]. Moreover, changes in the skin secretome profile have been reported in a number of models of skin-dysfunction, including ageing and psoriasis [23,24].

The skin is located adjacent to subcutaneous adipose tissue (sAT). sAT dysfunction plays a key role in the development of insulin resistance and T2D [15,[25], [26], [27], [28]]. sAT is the largest and safest lipid storage depot in the body [29]. However, due to its limited expansion capacity, the ability of sAT to store lipids can be exceeded, resulting in ectopic lipid deposition in metabolic organs such as the liver, skeletal muscle and pancreas [26], which can drive metabolic dysfunction and ultimately T2D [30]. Additionally, dysfunctional sAT secretes increased levels of pro-inflammatory cytokines and reduced levels of insulin sensitising adipokines and fatty acids [[31], [32], [33]], the combination of which further drives metabolic dysfunction.

Since lesional psoriatic skin is located directly adjacent to the sAT, we hypothesised that psoriatic skin inflammation and consequent alterations in the skin secretome could directly impact sAT function. Therefore, we have investigated whether skin-mediated sAT dysfunction constitutes a novel mechanism linking psoriasis with the development of comorbidities, including insulin resistance and T2D.

To investigate the role of the skin-sAT axis in psoriasis-mediated development of insulin resistance and T2D, we utilised *in vivo* and *ex vivo* mouse models and *ex vivo* human imiquimod (IMQ) models. IMQ is a member of the imidazoquinolamine class of compounds and has been found to stimulate an immune response largely through agonistic activity on toll-like-receptor (TLR)-7 and TLR8 signalling pathways [34,35]. The mouse IMQ model is one of the most reliable and routinely used animal models of psoriasis [[36], [37], [38], [39]], which has also been adapted for use in human *ex vivo* skin.

## 2 Materials and methods

### 2.1 Mouse IMQ-model

Animal procedures were conducted under the UK Home Office Animal Procedures (1986) Act. All work was approved by the King's College Animal Care and Ethics committee. C57Bl/6 mice (6–8 weeks old; Envigo, UK) were used for this study as this strain has been reported to present with a greater systemic inflammation in response to IMQ [35,36,40,41]. A 4 cm^2^ dorsal region was shaved and depilated using hair removal cream (Veet®, Slough, UK). A daily topical dose of 75 mg of 5% IMQ cream (3.75 mg IMQ/day; Aldara, Meda Pharma, UK) or Vaseline equivalent (control) was applied to the shaved dorsal region for 4 consecutive days [37]. Body weight, food intake, water intake, and imaging for observable changes were measured daily between 9 and 10 am. Double-fold skin thickness was also measured daily, with a mean of three readings taken each day using a thickness gauge with 0.01 mm graduation (OneCall, Farnell element14, Leeds, UK). On the 5th day, mice were either fasted for 6 h prior to the conduct of glucose or insulin tolerance tests or sacrificed in the fed state with tissues and serum collected.

### 2.2 Haemotoxylin and Eosin staining

Mouse skin sections (1 cm^2^) were fixed in 4% paraformaldehyde for 24 h prior to tissue processing (Leica TP 1020, Leica Biosystems, Germany) and embedding in paraffin wax. 5 μm paraffin sections were melted briefly on a hot plate before the slides were dewaxed using xylene and then rehydrated using solutions of decreasing ethanol gradient (100%–70%). The staining procedure was as follows: 5 min immersion in Haematoxylin, 5 min in running water, three to five dips in acid alcohol, 2 min in running water, five dips in Eosin and then 2 further minutes in running water. Following staining, the sections were dehydrated in an increasing ethanol gradient (70%–100%). Finally, the sections were immersed in xylene for 7 min before being mounted. Slides were imaged with a bright field channel on a microscope (Olympus BX40).

### 2.3 Human IMQ model

Human skin was obtained from corrective abdominoplasty surgery, with written patient consent. The East London and City Health Authority Research Ethics Committee approved the use of skin samples (09/HO704/69). sAT was removed and skin was washed in PBS before treatment with IMQ. 5% IMQ cream was applied topically to the skin (Aldara, Meda Pharma, UAE) using a method adapted from Fehres et al. (2014) [42]. Briefly, three sachets of Aldara cream were applied per 12 × 1 cm^2^ sections of skin (∼3.75 mg IMQ over 1 cm^2^). After 20 min of exposure to IMQ cream, excess cream was removed, and the skin was cleaned in sterile PBS before use in conditioned media experiments. Skin sections were incubated in serum-free DMEM (supplemented with l-glutamine and penicillin/streptomycin) at 37 °C and 5% CO2 in a humidified atmosphere for 24 h, following which tissue was snap frozen and CM collected.

### 2.4 Quantitative RT-PCR

Total RNA was extracted from ∼100 mg tissue, according to the manufacturer's instructions (RNeasy Lipid Mini Kit or RNeasy Mini Kit, Qiagen, UK). Gene expression was determined by Sybr Green qRT-PCR using ΔΔCt methodology, normalised against GAPDH (Quantitech, UK). Data are expressed relative to the average control treatment for each gene. Primer sequences (Eurogentec, Southampton, UK or Quantitech, UK) are shown in Supplemental Tables 1 and 2.

### 2.5 Intraperitoneal glucose and insulin tolerance tests (IPGTT & IPITT)

Mice were fasted for 6 h and then intraperitoneally administered 2 g/kg 30% glucose solution (for GTT) or 0.75 IU/kg insulin (for ITT). Blood glucose measurements were measured by tail prick over a 2-hour period using an Accucheck glucose meter (Roche Diagnostics, UK). Terminal blood samples were taken by cardiac puncture.

### 2.6 ELISA & Mesoscale U-Plex assays

Serum insulin and C-peptide were measure by specific ELISA (ALPCO, United States). Serum and CM cytokine measurements were measured using a U-PLEX TH17 Combo 2 kit (Meso Scale Diagnostics, United States).

### 2.7 Immunofluorescence

Paraffin embedded sections (5 μm) of mouse pancreas were incubated with polyclonal rabbit anti-Ki67 (Abcam, Cambridge, UK) and polyclonal guinea-pig anti-insulin (Agilent Pathology Solution, United States) primary antibodies (1:200). Secondary antibodies used were Alexa Fluor® 488 donkey anti-rabbit and Alexa Fluor® 594 donkey anti-guinea pig (1:200). DAPI was also used to stain the nuclei at a 1:1000 dilution (Sigma Aldrich, UK). Sections were mounted using Fluoromount Aqueous Mounting Medium (Dako, United States). InCell 2200 (GE Healthcare Life Sciences, UK) was used to acquire images and analysis (cell counting and islet area calculations) was performed using the GE InCell Developer Toolbox.

### 2.8 Mouse islet isolation

Mouse islets were isolated between 9 and 10 am [43]. Isolated islets were maintained in supplemented RPMI (10% FBS; 2 mM l-glutamine, 100 U/ml penicillin/0.1 mg/ml streptomycin) at 37 °C and 5% CO2 in a humidified atmosphere for up to 48 h until the experimental treatment.

### 2.9 Dynamic insulin secretion (perifusion)

Static or dynamic insulin secretion was assessed in response to 2 or 20 mmol/l glucose exposure, as previously described [44]. Secreted insulin was measured by in-house I125 radioimmunoassay [45].

### 2.10 Caspase-Glo® 3/7 apoptosis assay

Mouse islets were treated with conditioned media +/− cytokine cocktail (0.05 U/μl Il-1β, 1U/μl TNFα and IFNγ; 48 h). Apoptosis was determined by Caspase-Glo 3/7 luminescent assay (Promega, Southampton, UK).

### 2.11 Conditioned media (CM) experiments

For mouse CM experiments, skin obtained from IMQ- and Vaseline-mice on day 5 following sacrifice was incubated for 24 h in DMEM (containing 25 mmol/l glucose; 2 mmol/l glutamine, 10% FBS, 100U/ml penicillin, 100 μg/ml streptomycin). CM was collected after 24 h and combined 1:1 with fresh DMEM and used to treat control mouse sAT or islets for 24–48 h. For human CM experiments, skin-CM was subsequently diluted 1:1 in fresh supplemented DMEM and used to treat sAT (100 mg; obtained from abdominoplasty), or isolated mouse islets. After 24 h, CM was removed and sAT incubated for a further 24 h in fresh DMEM before tissue collection via snap freezing.

### 2.12 Data analysis

Data is expressed as mean ± SEM. Significance was tested using one or two-way ANOVA with Tukey's or Sidak's post-test, using GraphPad PRISM 7 software.

## 3 Results

### 3.1 Topical IMQ induced an inflammatory phenotype in mouse skin

**Figure 1:**
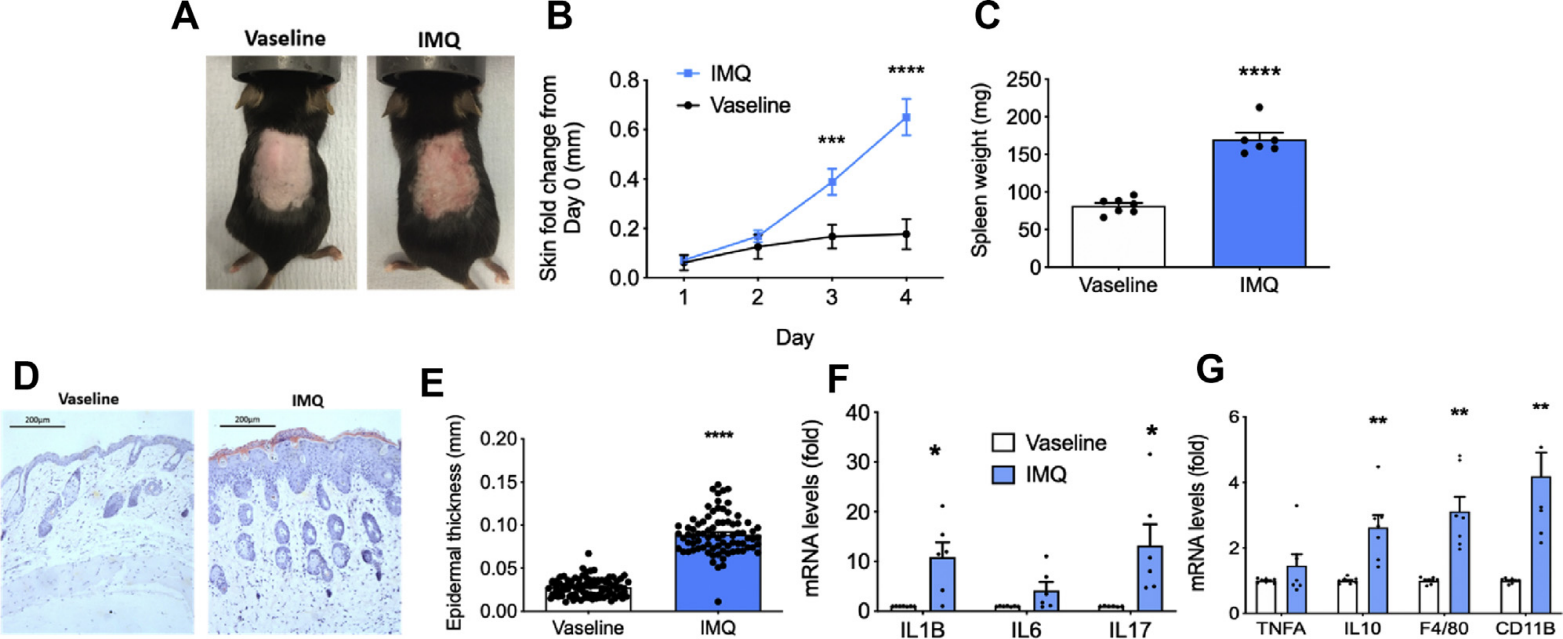
**IMQ induced an inflammatory phenotype in mouse skin**. C57Bl/6 mice were treated with IMQ (3.75 mg IMQ/day) or Vaseline control for 4 days. (A) Representative Day 4 images displaying erythema and scaling (B) daily double-fold skin thickness, n = 45, (C) post-sacrifice spleen weight (n = 6) and (D) Representative H&E staining of mouse skin. (E) Epidermal thickness of mouse skin calculated from H+E staining. 6 sections per mouse were stained and n = 3–4 mice were used. Four measurements were taken per image. (F–G) qPCR analysis of inflammatory gene expression in mouse skin, n = 6–8. Data is expressed as mean ± SEM. ∗P < 0.05, ∗∗P < 0.01, ∗∗∗P < 0.001, ∗∗∗∗P < 0.0001, vs. Vaseline control.

To examine the metabolic effects of skin inflammation, we utilised the IMQ-mouse model. Consistent with previous studies [36,37], IMQ treatment replicated many of the phenotypic changes observed in human psoriasis, including erythema and scaling (Figure 1A), increased skin-fold thickness (Figure 1B), splenomegaly (Figure 1C), and increased epidermal thickness, measured in skin sections taken post-sacrifice on Day 5 (Figure 1D+E). Moreover, gene expression of inflammatory markers (IL1β, IL6, TNFα, IL17 and IL10) were elevated in IMQ-mice compared to Vaseline controls (Figure 1F+G). In addition, gene expression of F4/80 and Cd11b were significantly increased in IMQ-mouse skin, indicating macrophage infiltration (Figure 1G). IMQ-mice showed a drop in body weight for the first 2 days of treatment; however, after 5 days, most of weight lost had been regained and there was little difference in weight between IMQ and the Vaseline treated controls (Supplementary Figure 1A). Similar trends were observed for food and water intake (Supplementary Figure 1B+C).

### 3.2 Topical IMQ application induces inflammation and markers of metabolic dysfunction

**Figure 2:**
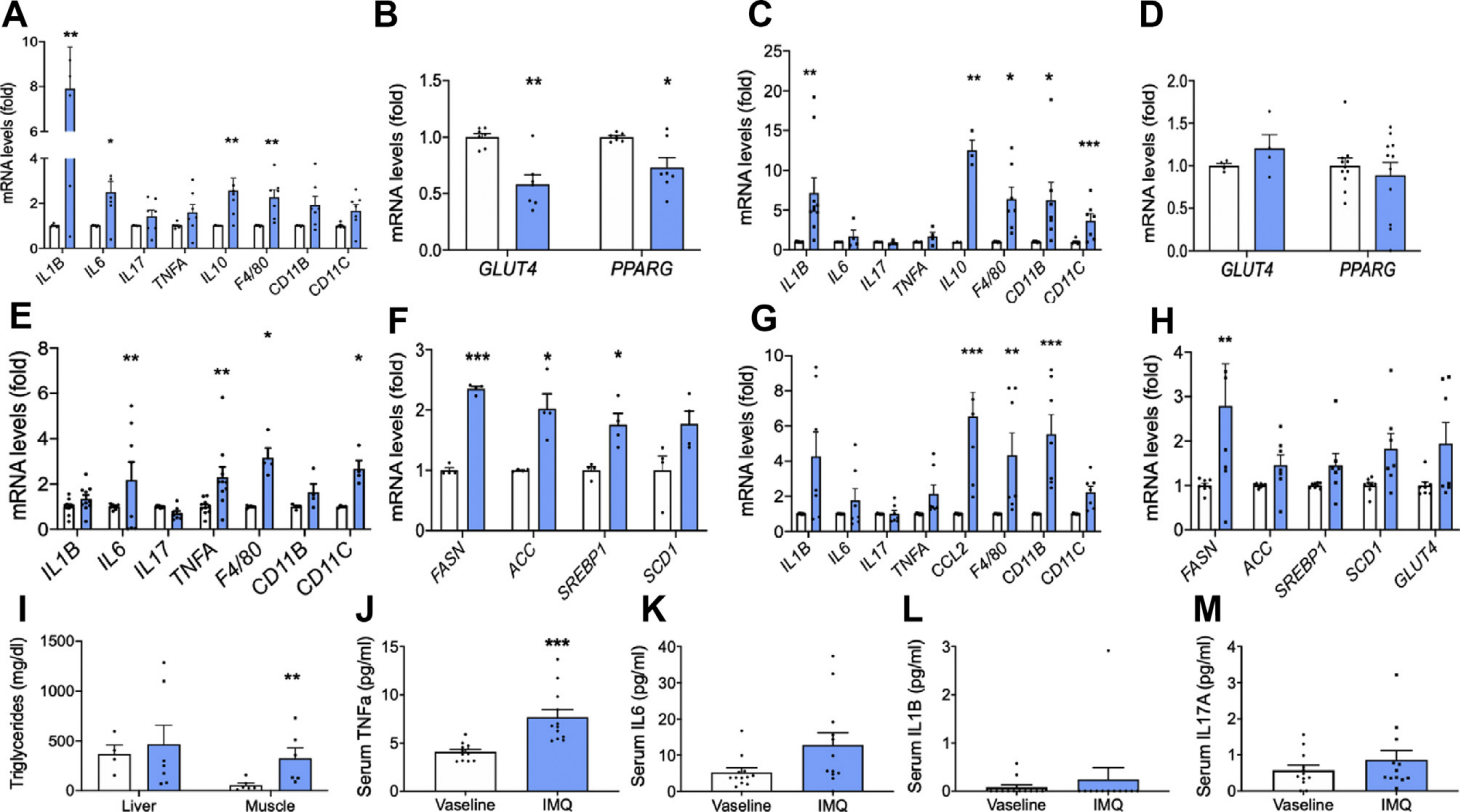
**Topical IMQ application induces inflammation and markers of metabolic dysfunction in mice.** Tissue and serum were obtained from C57Bl/6 mice treated with IMQ (3.75 mg IMQ/day) or Vaseline control for 4 days. **(A**–**H)** Gene expression of inflammatory and metabolic markers was assessed by qPCR in sAT (A–B), eAT (C–D), liver (E–F) and skeletal muscle (G–H), n = 4–10; (I) Triglyceride levels were assessed in liver and skeletal muscle by colorimetric assay; (J–M) Serum cytokine concentrations were measured by MSD U-plex assay; (J) TNFα, (K) IL6, (L) IL1β and (M) IL17A (n = 12). Black bars = Vaseline control, blue bars = IMQ. Data is expressed as mean fold change from controls ± SEM for gene expression and mean ± SEM for serum and tissue TG analysis. ∗P < 0.05, ∗∗P < 0.01, ∗∗∗P < 0.001 vs. Vaseline control.

We hypothesised that psoriatic skin inflammation could induce inflammation and metabolic dysfunction in key metabolic tissues. Given the proximity of skin to sAT, we initially assessed whether skin inflammation could affect sAT function through the measurement of changes in key inflammatory and metabolic genes. Consistent with this hypothesis, an inflammatory phenotype was observed in the sAT, denoted by significant increases in IL1B, IL6 and IL10 gene expression in IMQ-mice compared to Vaseline-treated controls, as well as a significant increase in the expression of the macrophage marker F4/80 (Figure 2A). Additionally, trends towards increased gene expression of TNFA, IL17A, CD11B (a marker for macrophages and microglia) and Cd11c (a dendritic cell marker) in the IMQ-treated mice were observed (Figure 2A). Conversely, gene expression of the insulin sensitising, pro-adipogenic nuclear receptor PPARG was significantly decreased in IMQ-mouse sAT. Likewise, sAT gene expression of GLUT4, a critical protein for insulin-stimulated glucose uptake, was also significantly decreased (Figure 2B).

To examine the effects of skin inflammation in more distal metabolic tissues, we assessed the expression of metabolic and pro-inflammatory markers in epididymal adipose tissue (eAT), liver, skeletal muscle and gut tissue obtained from IMQ mice. Gene expression of inflammatory markers (IL1B, IL10, F4/80, CD11C) was significantly elevated in eAT (Figure 2C), although unlike sAT, these changes were not associated with reduced GLUT4 or PPARG (Figure 2D). Moreover, IMQ induced liver and skeletal muscle inflammation, characterised by significant increases in liver IL6, TNFA, F4/80 and CD11C mRNA (Figure 2E) and muscle IL1B, CCL2, F4/80 and CD11B mRNA (Figure 2G) in IMQ mice compared to Vaseline control. These changes were associated with increases in expression of liver and muscle lipogenic genes (FASN, ACC, SREBP1, SCD1; Figure 2F,H) and marked elevation in muscle triglyceride levels (Figure 2I). Topical IMQ also induced significant increases in inflammatory markers in gut tissue (Supplementary Figure 2). IMQ-treated mice displayed trends towards a systemic inflammatory phenotype, with significantly increased serum levels of TNFα (P < 0.001; Figure 2J) and trends towards increased IL6, IL1B, IL17A (Figure 2K–M). Serum fatty acid and triglyceride levels were unchanged (data not shown). Together, these data demonstrate the skin inflammation can induce tissue and systemic inflammation and signs of metabolic dysfunction in key metabolic tissues.

### 3.3 IMQ mediates changes in whole-body glucose tolerance and β-cell function

**Figure 3:**
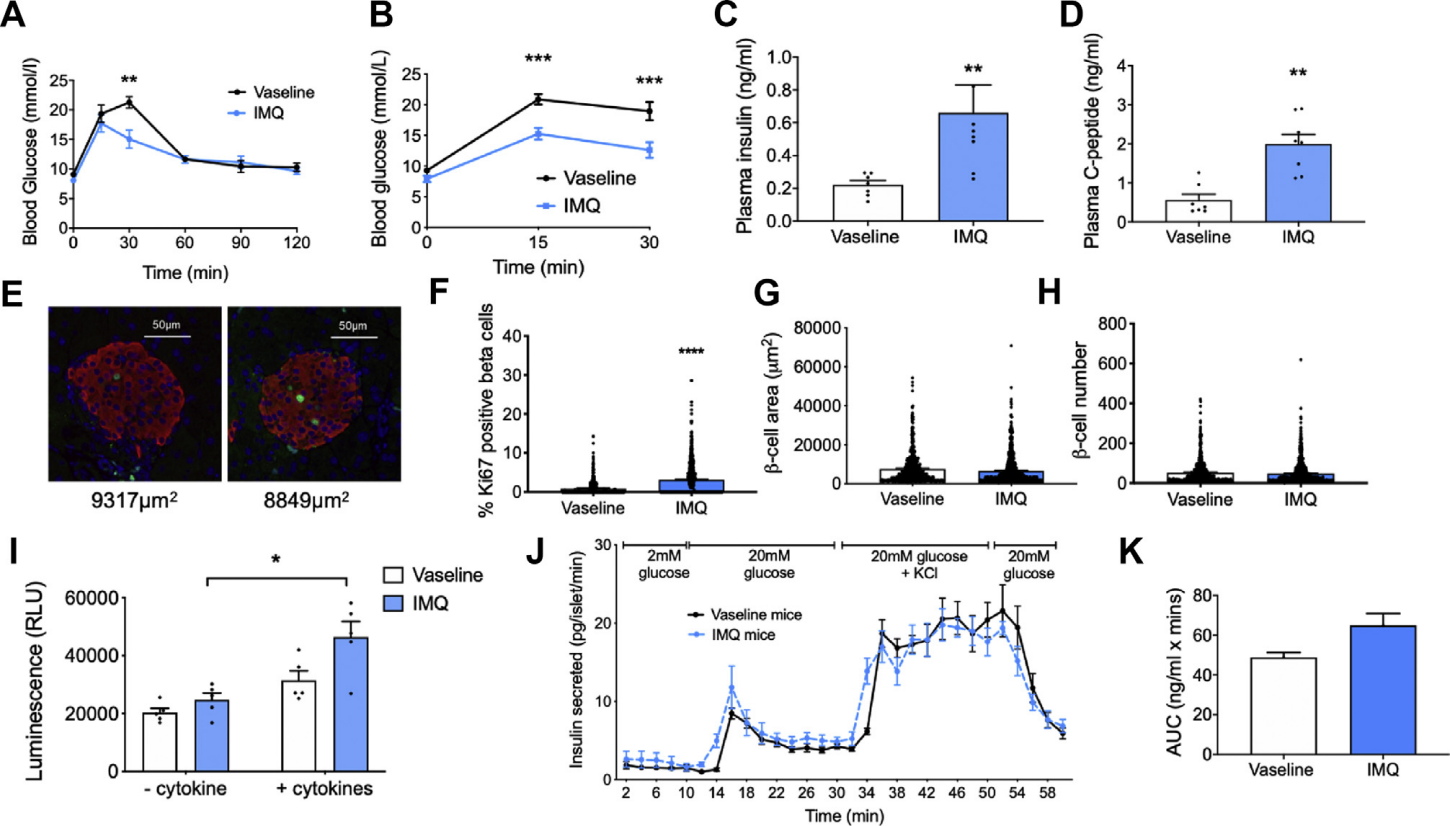
**Glucose tolerance and β-cell proliferation are enhanced in IMQ mice.** C57Bl/6 mice were treated with IMQ (3.75 mg IMQ/day) or Vaseline control for 4 days. Glucose response to IPGTT over (A) 2-hours and (B) 30 min. (C) Plasma insulin and (D) plasma C-peptide levels from terminal blood samples taken at 30 min post-glucose administration, (n = 6–7). (E) Representative immunofluorescence images of pancreata stained for insulin (red), Ki67 (green) and DAPI (blue) (F) percentage of Ki67 positive β-cells, (G) β-cell area, and (H) β-cell number, n = 7. (I) Caspase-Glo® 3/7 apoptosis assay using IMQ- and Vaseline-mouse islets, n = 4–5. (J) Dynamic glucose-stimulated insulin secretion using isolated islets from IMQ- and Vaseline-mice (four channels were used per treatment using islets pooled from six mice for each experiment, results are pooled from n = 2 experiments), and (K) area under the curve for the first 30 min, n = 5. Data is expressed as mean ± SEM. ∗P < 0.05, ∗∗P < 0.01, ∗∗P < 0.01, ∗∗∗P < 0.001.

Since IMQ treatment induced tissue and serum changes consistent with metabolic dysfunction, we next examined the effects of topical IMQ application on whole-body glucose regulation, using glucose tolerance tests. Fasting blood glucose (time 0) levels were unchanged between IMQ- and Vaseline-treated mice at day 5. However, during the IPGTT, we observed a significant reduction in blood glucose in the IMQ mice 30 min post-glucose bolus (Figure 3A; P < 0.01). Following this, 30-minute IPGTTs were carried out with terminal blood taken at the end time-point. During these shorter GTTs, the decrease in blood glucose for 30 min post-glucose bolus was even more striking (Figure 3B; P < 0.001) and terminal blood analysis demonstrated significant increases in plasma insulin and C-peptide (Figure 3C+D; P < 0.01), indicative of increased pancreatic β-cell insulin secretion. We next analysed in greater depth whether changes in glucose tolerance might be linked to altered beta-cell functional mass. Immunofluorescence staining of IMQ- and Vaseline-treated mouse pancreas for insulin and the proliferative marker Ki67 revealed a significant increase in the percentage of Ki67 positive β-cells in IMQ-mice pancreas Figure 3E–H). These changes were accompanied by, a significant increase in cytokine-mediated apoptosis in IMQ-mouse islets (Figure 3I). Simultaneous apoptosis and proliferation are consistent with reports of beta-cell mass compensation during T1D disease progression [46,47]. Islet area and β-cell number were unchanged between the IMQ and Vaseline groups (Figure 3G+H). Moreover, isolated IMQ-mouse islets were characterised by a quicker response time to changes in glucose concentration and a trend towards increased area under the curve for the first 30 min, suggestive of a small but non-significant increase in 1st-phase insulin secretion (Figure 3J+K).

Recent studies have reported that IMQ is present in serum at concentrations of ∼75 ng/ml up to 2 days after topical application [48]. To assess whether the effects observed in Figure 2, Figure 3 could be mediated by IMQ directly, rather than through skin inflammation, we exposed isolated mouse islets and sAT to 75 ng/ml IMQ for 24 h (Supplementary Figure 4). However, direct IMQ treatments had no effect on sAT gene expression isolated from CD1 and C57Bl/6 mice, nor on insulin secretion from islets isolated from CD1 mice. Direct IMQ treatment did cause a trend towards reduction in insulin secretion from C57Bl/6 islets. However, these findings of reduced insulin secretion with direct IMQ do not reflect the *in vivo* data, in which insulin secretion was increased, so we remain confident that the effects observed *in vivo* in the IMQ model did not occur via direct effects of absorbed IMQ.

Together, this data suggests that psoriatic skin inflammation mediates multiple effects on whole-body glucose regulation and key metabolic tissues. These effects were unlikely to be mediated by direct effects of absorbed IMQ, indicating a potential role for alterations in the skin secretome in mediating metabolic effects of skin inflammation.

### 3.4 Skin-derived factors induce sAT inflammation following IMQ treatment of mouse and human skin

Since skin is located adjacent to sAT, we hypothesised that skin-derived factors could particularly influence sAT function. To determine whether changes in sAT inflammation in the IMQ model occurred via the actions of the skin secretome, we conducted conditioned media (CM) experiments using mouse and human skin.

**Figure 4:**
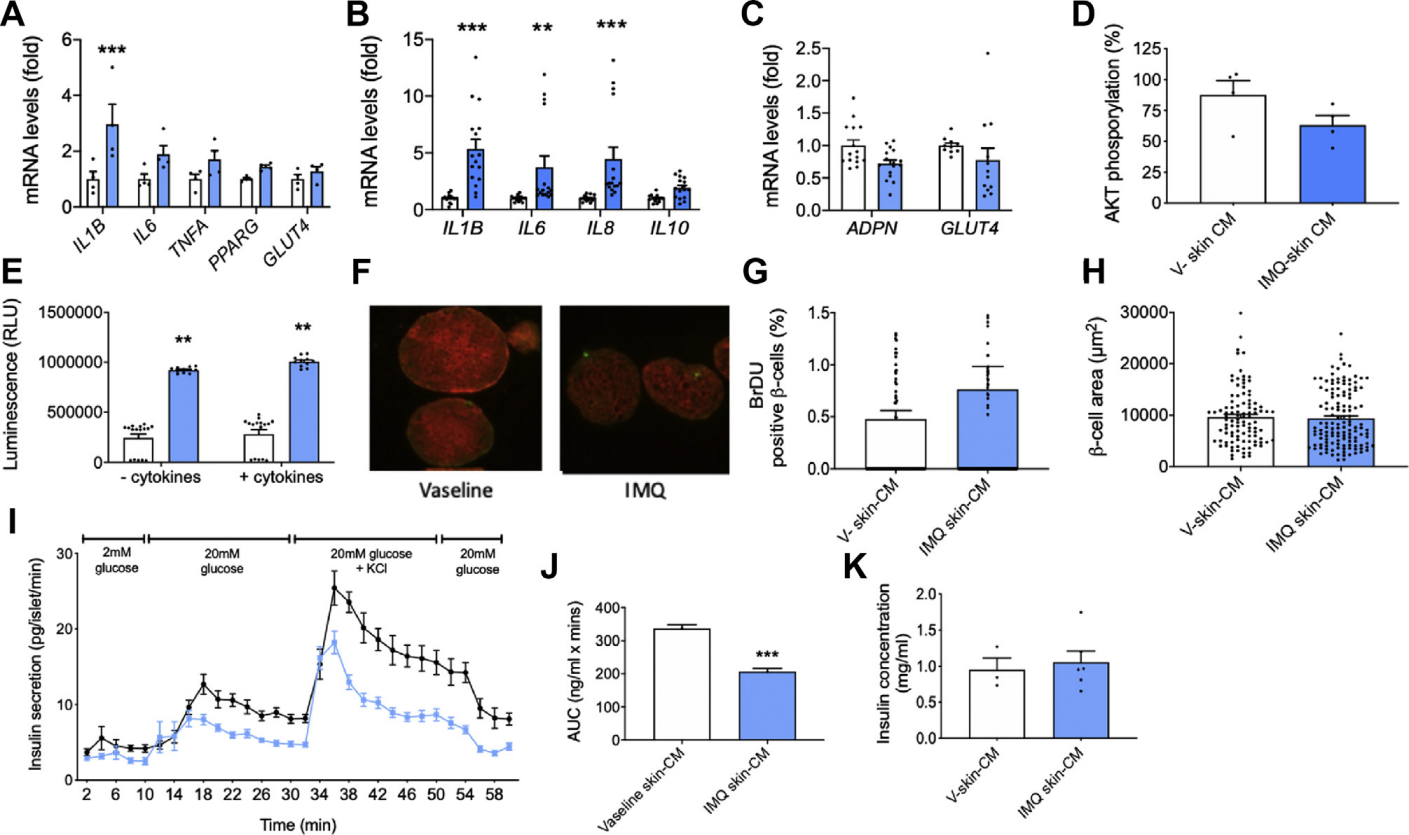
**Effects of skin-derived factors on sAT inflammation and dysfunction and pancreatic beta-cell functional mass.** Mouse sAT was incubated in mouse skin CM for 48 h. Human sAT was incubated in human skin CM for 24 h, and then in serum-free DMEM for a further 24 h. Gene expression of inflammatory and metabolic markers was assessed by qPCR in (A) mouse sAT and (B–C) human sAT. (D) Phospho(Ser47) AKT levels were assessed using a whole-cell lysate kit (Mesoscale Discovery) in human sAT. For mouse sAT, n = 4 mice. For human results n = 4 collections of sAT from four different donors. (E–K) Mouse islets were incubated for 24 h with skin CM collected from cultured Vaseline or IMQ treated mouse skin. (E) Islet apoptosis, n = 11; (F–H) Beta-cell BrDU uptake, 150–200 islets pooled from six mice were used for each treatment group. (F) Islets were stained for insulin (red) and BrDU (green); (G) percentage of BrDU positive β-cells and (H) β-cell area. Representative images for each treatment group are also shown (F). (I) Dynamic glucose-stimulated insulin secretion using isolated islets treated with IMQ-mouse skin CM for 24 h. Four channels were used per treatment using islets pooled from six mice. Perifusion results are presented from two pooled experiments (J) Area under the curve calculated from (I). (K) Islet insulin content, n = 3–6 replicates per treatment group. Data is expressed as mean ± SEM. White bars = Vaseline control, blue bars = IMQ. Data is expressed as mean fold change vs controls ± SEM. ∗∗P < 0.01, ∗∗∗P < 0.001 vs Control.

Skin was collected from the IMQ- and Vaseline-mice post-sacrifice at day 5 and cultured for 24 h sAT collected from healthy control mice was incubated with CM obtained from cultured mouse skin, to determine the effects of the skin secretome on healthy sAT. IMQ-skin-CM induced a significant increase in sAT gene expression of IL1B (P < 0.001) and non-significant increases in IL6 and TNFA (Figure 4A), suggesting that sAT inflammation in IMQ mice is possibly driven by actions of the skin secretome. Of note, since the skin samples used to generate CM were taken from the lesional site of IMQ application, it is possible that the systemic inflammatory effects of skin inflammation could impact back on the skin, e.g., via cytokine release from inflamed sAT, prior to skin sampling for *ex vivo* studies.

Next, to examine whether these effects were translatable to human skin and sAT, we induced a psoriatic phenotype in human skin *ex vivo* via exposure to IMQ. Exposure of human skin to IMQ induced a pro-inflammatory phenotype, similar to that observed in IMQ-mouse skin, characterised by significant increases in IL1B, IL6 and IL8 mRNA (Supplementary Figure 4). Skin CM obtained following culture of human skin for 24 h, was then used to incubate human sAT *ex vivo*. Exposure to IMQ-skin-CM led to significant increases in sAT levels of IL1B, IL6 and IL8 (all P < 0.001) and non-significant decreases in *ADPN* and *GLUT4* compared to control-skin-CM (Figure 4B–C), although there was some patient-to-patient variation within these changes. In human sAT, IMQ-skin-CM exposure also led to small decrease in sAT levels of phospho(Ser73)AKT, a marker of insulin signalling (Figure 4D).

Together, these data suggest that skin inflammation may be able to induce sAT dysfunction via the actions of the skin secretome in both mouse and human experiments.

### 3.5 Skin-derived factors exert mild effects on pancreatic islet functional mass

Since beta-cell proliferation and apoptosis were altered *in vivo* in IMQ-treated mice, we next examined the effects of the skin secretome on isolated pancreatic islets. Mouse skin-CM was obtained as described in 3.4 and used to treat isolated mouse pancreatic islets for 24 h. Incubation of mouse islets with mouse skin-CM partially mimicked the islet phenotype observed in IMQ mice *in vivo*. IMQ-skin-CM significantly increased islet apoptosis (Figure 4E), whilst there was a small but non-significant increase in beta-cell proliferation (as measured by BrDU uptake) compared to CON-skin-CM (Figure 4F–G). However, in contrast to the *in vivo* situation, IMQ-skin-CM reduced dynamic islet insulin secretion and insulin content (Figure 4I–K).

Finally, we conducted preliminary analysis of the skin secretome using a U-Plex multiplex ELISA to measure cytokine levels in Vaseline and IMQ mouse CM. This analysis demonstrated that IL17A and IL1β levels were non-significantly increased in IMQ-CM compared to Vaseline-CM, whilst IL6 and TNFα were non-significantly reduced in IMQ-CM compared to Vaseline-CM (Supplementary Figure 5).

Together, these data suggest changes in the skin secretome occurring in response to psoriatic skin inflammation may induce some of the *in vivo* changes in beta-cell functional mass observed in IMQ mice.

## 4 Discussion

Psoriasis is reported to be an independent risk-factor for the development of insulin resistance and T2D [[4], [5], [6], [7], [8]]. This, together with studies showing that skin-specific transgenic mouse models display altered whole-body glucose metabolism, suggests a direct role for the skin, and potentially the skin-secretome, in mediating increased risk of T2D in psoriasis [9,10]. However, the effects of skin inflammation and the skin secretome, on glucose homeostasis are poorly characterised. Here, using a combination of murine and human experimental models, we provide evidence that psoriatic skin inflammation induces multiple metabolic changes consistent with development of pre-diabetic phenotype, potentially via the actions of the skin secretome.

Specifically, IMQ-induced psoriatic-skin inflammation induced systemic inflammation with increased markers of inflammation, insulin resistance and dyslipidaemia in key metabolic tissues including AT, liver, muscle and gut. Consistent with onset of a pre-diabetic phenotype, the mice simultaneously displayed signs of islet compensation, including increased glucose-stimulated insulin secretion, beta-cell proliferation and improved glucose tolerance.

Importantly, we demonstrated a likely role for the skin secretome in driving sAT dysfunction observed in IMQ mice. In both mouse and human models, incubation of sAT with skin-CM partly recapitulated the inflammatory and metabolic effects observed in sAT *in vivo*. This suggests that the effects observed *in vivo* potentially occur via the actions of factors secreted from the skin, although additional experiments in which human sAT is exposed to IMQ alone are required to rule out a direct effect of IMQ. These effects are consistent with reports that UV light exposure, as a model of skin ageing, leads to alteration of skin pro-inflammatory cytokine secretion, which in turn modulates sAT function [49,50]. Other studies have reported altered sAT expression of cholesterol efflux regulating miRNAs in biopsies taken from directly below psoriatic lesions compared to sAT taken from beneath non-lesional or healthy skin [51].

The ability of the skin secretome to induce sAT dysfunction likely plays a key role in driving IMQ-mediated changes in whole-body glucose homeostasis via sAT inflammation and reductions in PPARG and GLUT4 mRNA. PPARG is an anti-inflammatory, insulin sensitizing nuclear receptor, which also plays a key role in adipogenesis [52]. Reductions in PPARG may be linked to induction of systemic inflammation as well as ectopic lipid deposition into skeletal muscle and liver. GLUT4 encodes for insulin-regulated glucose transporter 4. Although sAT is only responsible for ∼10% of prandial glucose uptake, sAT GLUT4 levels strongly correlate with insulin sensitivity, since AT GLUT4 employs a disproportionate control over whole-body glucose tolerance [29,53]. Therefore, IMQ-mediated reductions in sAT GLUT4 are potentially causal for changes in whole-body glucose homeostasis.

Whilst broadly consistent data was observed across the mouse and human skin models, there are numerous differences between the models. A key difference is the duration of IMQ exposure, in that any effects resulting from 20 min IMQ exposure and 24 h culture in the human skin model would likely only occur due to immediate TLR7/8 agonism [40]. For example, IMQ can directly agonise TLR7/8 on keratinocytes, leading to upregulation of pro-inflammatory cytokines, including IL8 and TNFα, via NFκB mediated mechanisms, whilst TLR7/8 are also present on Langerhan cells [54,55].

In contrast, 4-day IMQ exposure in the *in vivo* model would likely induce additional T-cell mediated mechanisms and infiltration of other immune cell types [40]. Such differences in the mechanisms of action may ultimately impact downstream mechanisms and outcomes. Future studies will investigate these differences in more depth. It will also be of interest to investigate the effects of longer IMQ exposure in human skin models, although such experiments are constrained by the limitations of *ex vivo* skin culture in which skin typically begins to lose integrity after 24 h.

**Figure 5:**
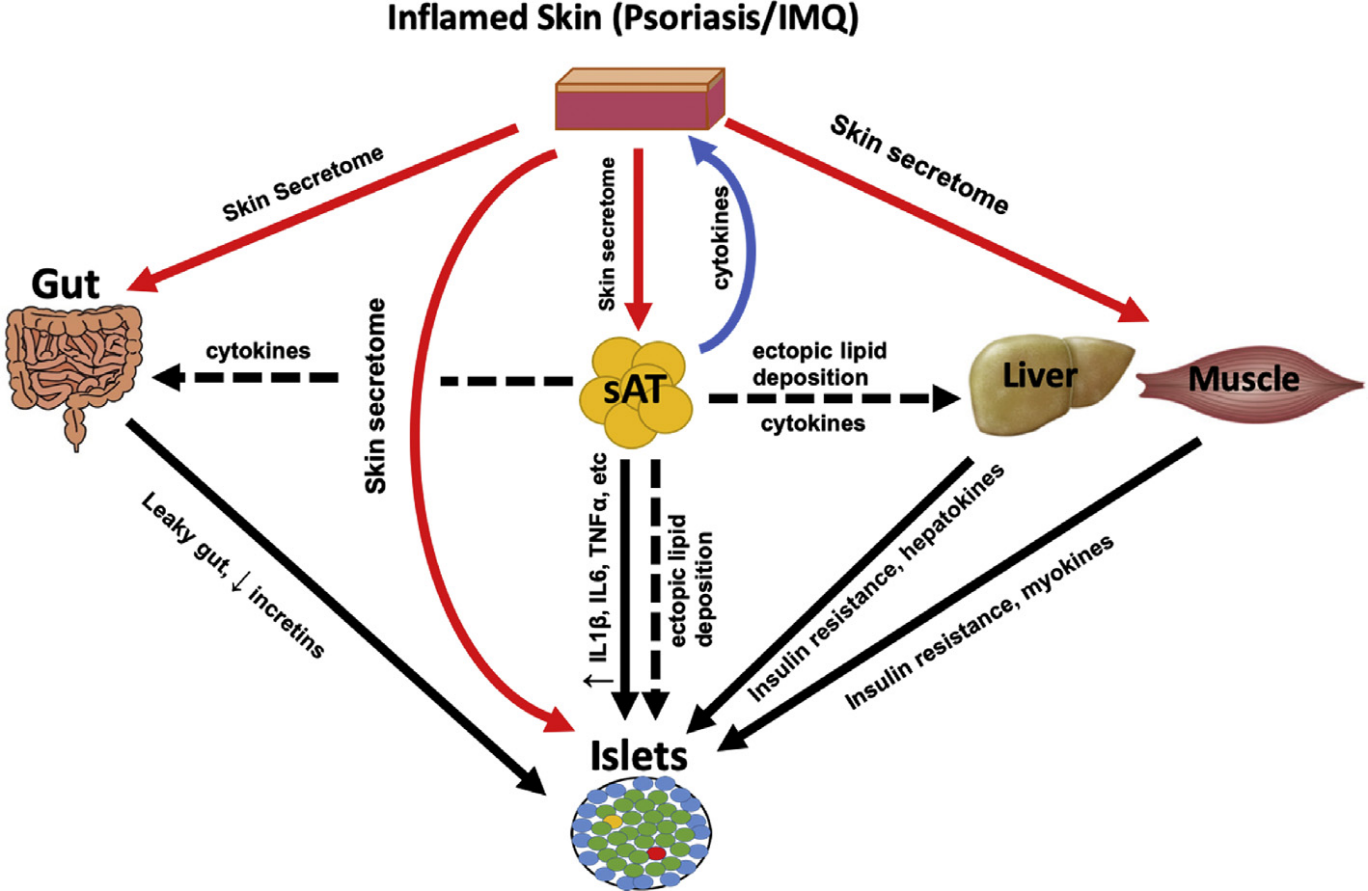
Schematic of proposed hypothesis showing how skin inflammation contributes to whole-body glucose metabolism.

The psoriatic-skin secretome was also potentially responsible for observed changes in pancreatic islet functional mass, with mild increases in beta-cell proliferation and apoptosis. However, IMQ-skin-CM only recapitulated some of the *in vivo* beta-cell changes. This suggests that factors other than those derived from the skin mediate beta-cell changes in the IMQ model. For example, a wide range of bioactive proteins are secreted from adipose tissue, liver, skeletal muscle and gut, whilst pancreatic islet hormones themselves can exert autocrine and paracrine effects on the beta-cell. Since IMQ induced inflammation and metabolic dysfunction in these tissues, secretion of bioactive factors from these tissues may also change, potentially explaining some of the observed changes in beta-cell functional mass, which were not accounted for by the skin secretome (Figure 5).

Importantly, psoriatic skin has been shown to display an altered secretome [23]. Whilst our data suggest a role for the skin secretome in mediating the systemic effects of skin inflammation, the identity of the key skin-derived factors which mediated these effects is unclear. Our studies have identified IL1β and IL17A as cytokines which are potentially increased in IMQ-skin CM suggesting these could play a role in the observed phenotype. Many other inflammatory cytokines and chemokines are reportedly secreted from sebocytes, keratinocytes and immune cells within the skin, including, but not limited to, IL1β, IL6, IL8, TNFα, eNAMPT, adiponectin and glucocorticoids [3,[11], [12], [13], [14], [15], [16]]. All of these factors have established endocrine metabolic functions, hence alterations in their secretion levels from skin would likely induce systemic metabolic alterations [[17], [18], [19], [20], [21], [22]]. For example, pro-inflammatory cytokines, including IL1β and IL17A, can induce sAT dysfunction, leading to poor lipid storage, insulin resistance and inflammation. The effects of pro-inflammatory cytokines on islets are more complex. Although often assumed to be deleterious to β-cell function, inflammation can also exert beneficial effects on the pancreatic beta-cells functional mass. For example, low concentrations of IL1β have been shown to induce increased β-cell proliferation and insulin secretion, whereas long-term IL1β exposure has a negative impact on islet and β-cell function [17,20,[56], [57], [58], [59]]. IL6 has also been shown under some circumstances to stimulate insulin secretion [18], whilst subsets of islet macrophages have been reported to simultaneously impair insulin secretion but promote beta-cell proliferation in models of insulin resistance [60]. Future studies in which elements of the skin secretome, such as IL1β and IL17A, are blocked *in vitro* and *in vivo* are now required to definitively demonstrate the importance of these factors in inducing the phenotype observed in response to IMQ.

## 5 Conclusion

In summary, we have shown that an IMQ-induced psoriatic skin-inflammation can mediate systemic dysfunction in key metabolic tissues including sAT and pancreatic islets, as well as whole-body changes in glucose homeostasis. These changes potentially occur, in part, due to changes in the skin secretome, supporting the hypothesis that the endocrine function of skin could play an important, previously overlooked role in whole-body metabolic homeostasis (Figure 5). Dysfunction of this regulatory process may be a key mediator of psoriasis co-morbidities, particularly T2D. Treatment of skin inflammation and improvement of skin health could potentially be used to treat or prevent psoriasis co-morbidities. Identifying the specific skin-derived factors responsible will be crucial to understanding the inter-organ cross talk which links psoriasis and T2D. Specific skin-derived factors could potentially be used as novel biomarkers for predicting the risk of co-morbidity development in psoriasis patients.

## Author contributions

EAE, SRS, XK, YX, MS, AR, JF, SDB, MPP, RFH and PWC designed and conducted research, analysed data and reviewed and approved the final manuscript; EAE and PWC wrote the paper. PWC is the guarantor of this work. Data are available on request from the authors.
