# Supplementary material for: Psoriatic skin inflammation induces a pre-diabetic phenotype via the endocrine actions of skin secretome
Source: Mol Metab. 2020 Jun 26;41:101047. doi: 10.1016/j.molmet.2020.101047 (PMC7452265; doi:10.1016/j.molmet.2020.101047)
Supplement: Multimedia component 1 [file mmc1.docx]

**
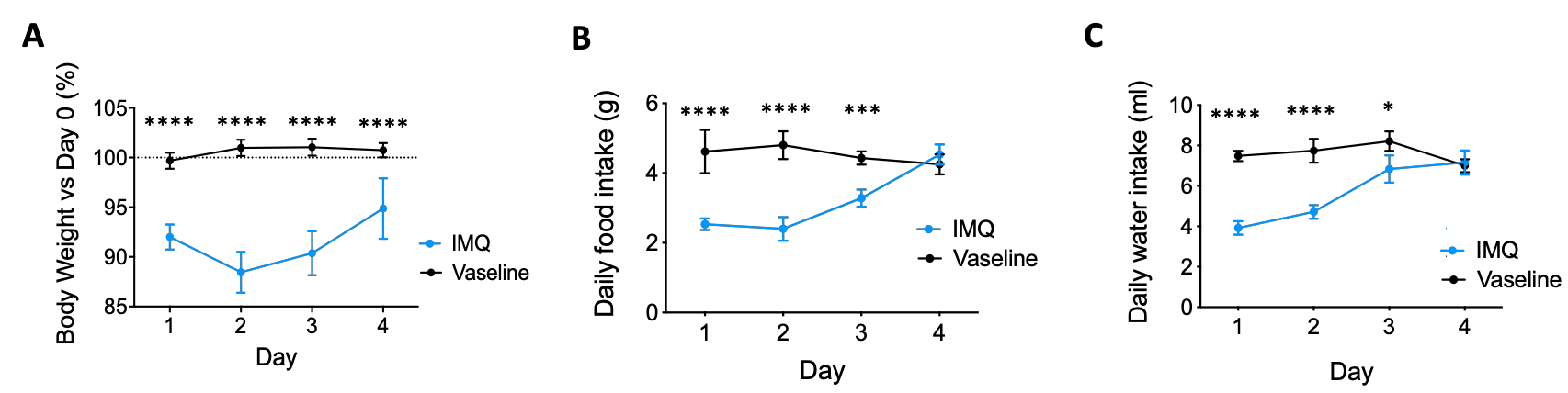
**

**Supplementary Figure 1 – Daily measurements in IMQ and Vaseline mice.** During the 5 days of IMQ treatment (Day 0 – 4), (A) body weight; (B) food intake and (C) water intake were measured between 9 – 10 am each day, n = 44-46. Data is expressed as mean ± SEM. *P<0.05, ***P<0.001, ****P<0.0001.


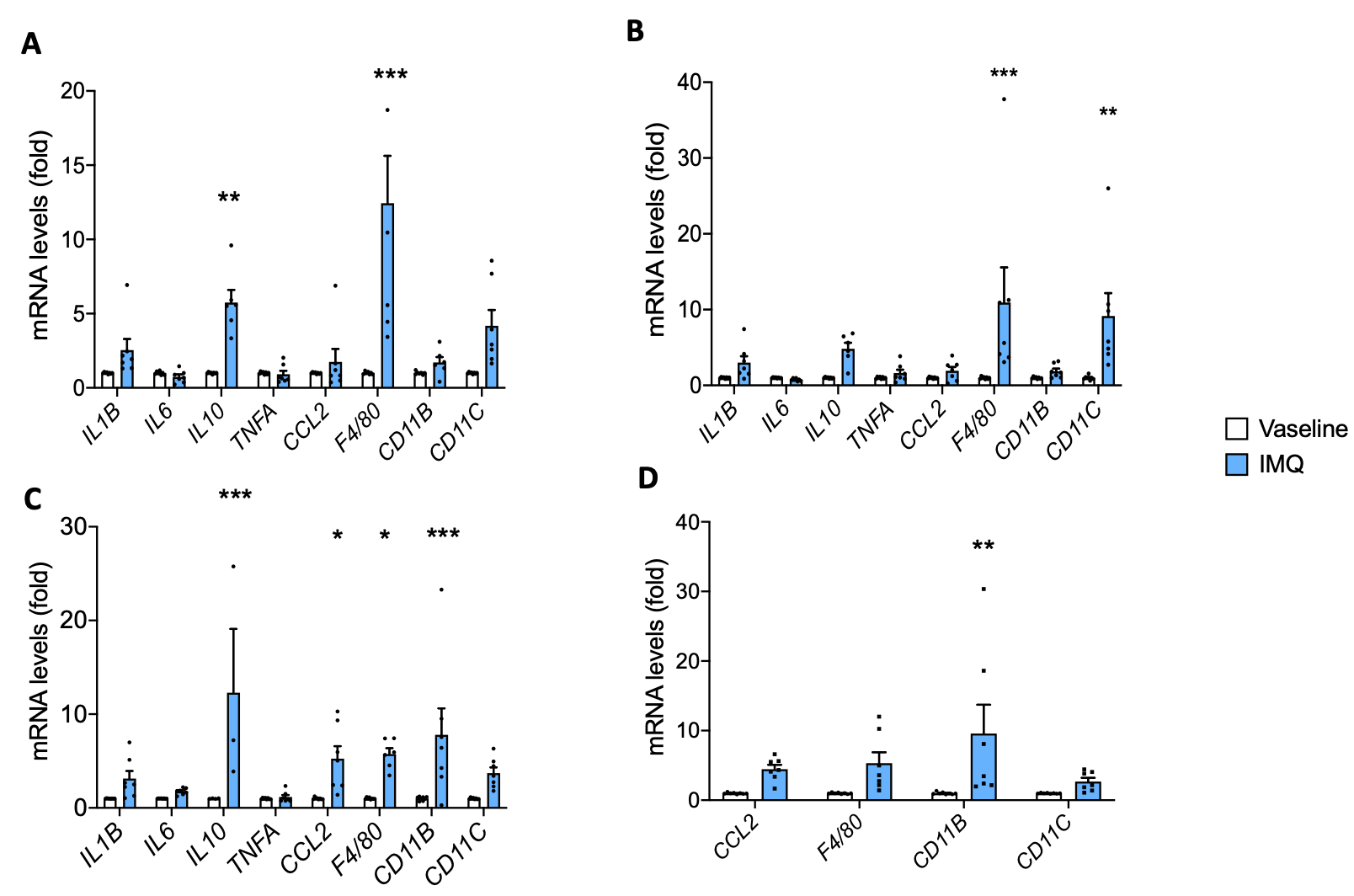


**Supplementary Figure 2 – Topical IMQ application induces gut inflammation in mice.** Gut tissue sections were obtained from 4-day IMQ (3.75 mg IMQ/day) or Vaseline treated mice. **(A – D)** Gene expression of inflammatory markers was assessed by qPCR in (A) jejunum, (B) duodenum, (C), ileum and (D) colon (n = 4-10). Data is expressed as mean fold change from controls ± SEM *P<0.05, **P<0.01, ***P<0.001 vs. Vaseline control.


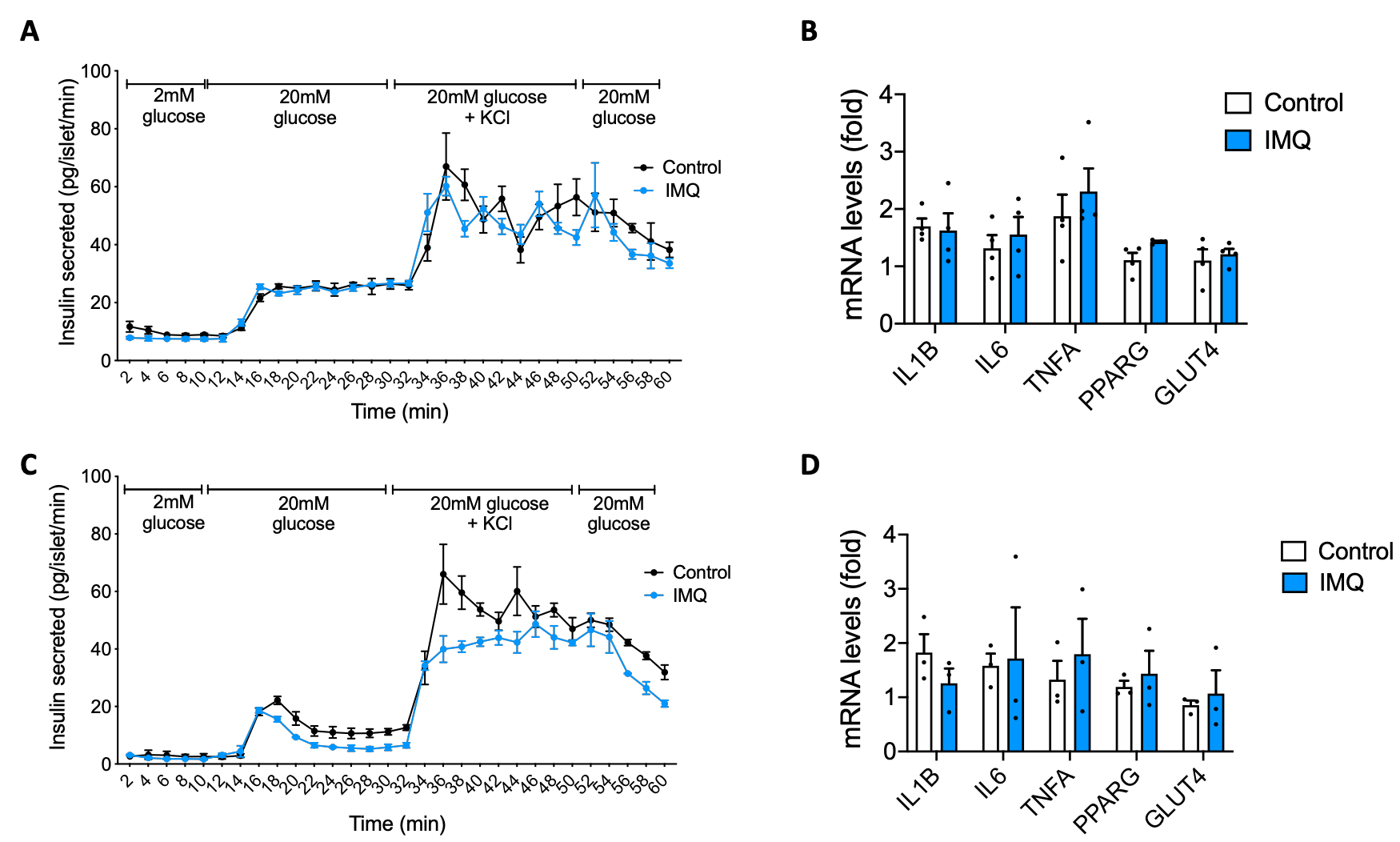


**Supplementary Figure 3 –** Direct treatment of sAT and islets with IMQ. Islets and sAT isolated from CD1 or C57Bl/6 mice were directly incubated with IMQ (75 ng/ml). The direct effect of IMQ treatment on dynamic glucose-stimulated insulin secretion in CD1 (A) and C57/B6 (C) mouse islets. Gene expression of key pro-inflammatory, adipogenic and glucose regulatory genes was also measured in sAT from CD1 (B) and C57/B6 (D) mice. For islet experiments, four channels were used per treatment using islets pooled from six mice. For sAT experiments n = 3-4. Data is expressed as mean ± SEM.

**
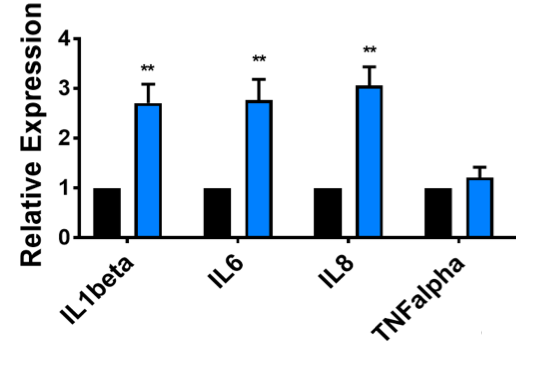
**

**Supplementary Figure 4 – Gene expression analysis of whole skin tissue treated with IMQ cream (5% IMQ).** Genes tested were as follows: IL1B, IL6, IL8 and TNFA. Skin analysed was treated with Aldara cream for 20 minutes and then cultured for 24 hours, n = 5-7 patients. Black bars = Vaseline control, blue bars = IMQ. Data is expressed as mean ± SEM. **P<0.01.

**
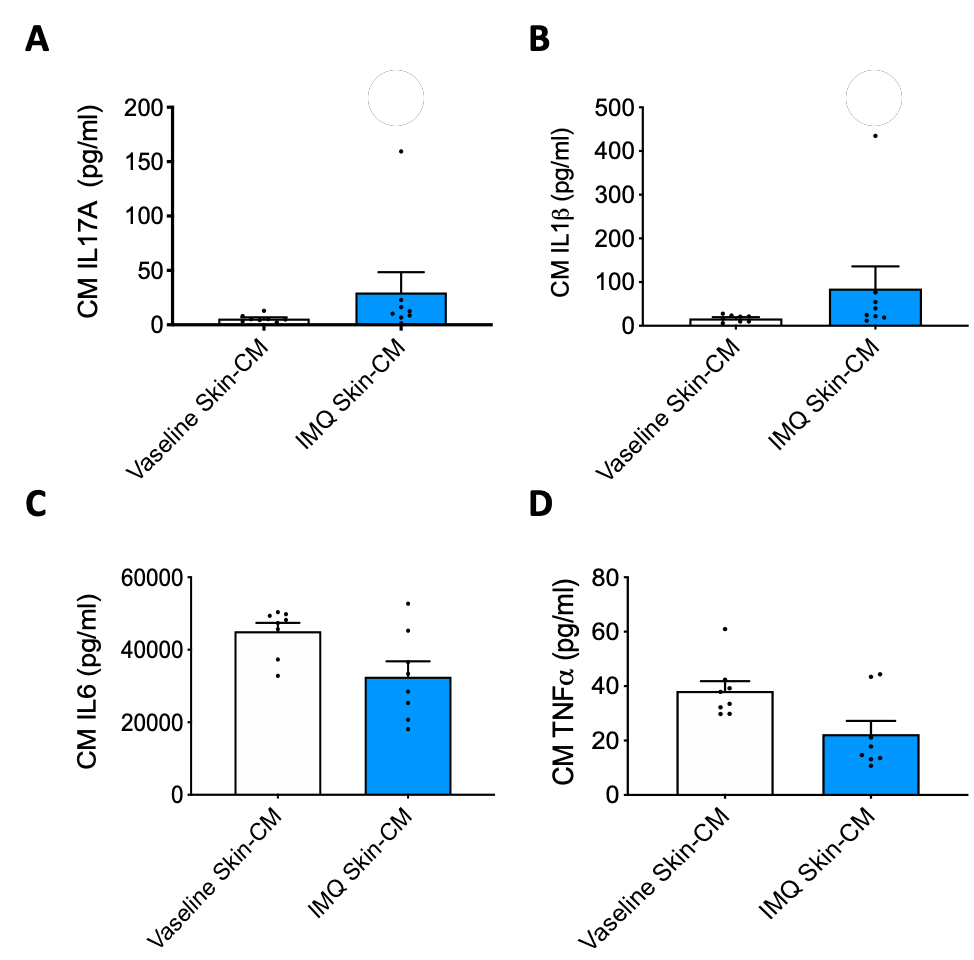
**

**Supplementary Figure 5 –** Skin was collected from Vaseline and IMQ mice, cultured for 48 hours with conditioned media collected. Cytokine levels were assessed in conditioned media using a U-Plex multiplex ELISA (Mesoscale Discovery). (A) IL17A (B) IL1β (C) IL6 (D) TNFα. n = 8 Data is expressed as mean ± SEM.

**Supplementary tables**
